# Influence of Vitamin D and C on Bone Marrow Edema Syndrome—A Scoping Review of the Literature

**DOI:** 10.3390/jcm11226820

**Published:** 2022-11-18

**Authors:** Annette Eidmann, Marius Eisert, Maximilian Rudert, Ioannis Stratos

**Affiliations:** Department of Orthopaedic Surgery, Koenig-Ludwig-Haus, Julius-Maximilians University Wuerzburg, Brettreichstrasse 11, 97074 Wuerzburg, Germany

**Keywords:** lower extremity, regional transient osteoporosis, bone marrow edema, vitamin D, vitamin C, scoping review

## Abstract

Bone marrow edema syndrome (BMES) is a rare disease with a largely unknown etiology. The aim of this scoping review is to systematically evaluate and combine the available evidence about vitamin D and C and BMES. The analysis of the manuscripts was based on country of origin, number of patients, gender, study type, epidemiology, localization, bone mineral density measurements, vitamin status and therapy. Sixty studies were included. The overall number of patients was 823 with a male-to-female ratio of 1.55:1 and a mean age of 40.9 years. Studies were very heterogeneous and of diverging scientific scope with a weak level of evidence. The hip was the most affected joint, followed by the foot and ankle and the knee; 18.3% of patients suffered from multifocal BMES. Sixteen studies reported on vitamin D levels, resulting in a high prevalence of vitamin D deficiency (47%) and insufficiency (17.9%) among BMES patients. Three BME manuscripts were associated with vitamin C deficiency. Current therapeutic interventions include conservative measures (mainly unloading), various osteoactive drugs and iloprost. In summary, data about BMES in association with vitamin status is limited. A causal relationship between vitamin D or vitamin C status, osteopenia, and BMES cannot be determined from the existing literature.

## 1. Introduction

The term “bone marrow edema” (BME) describes an MR tomographic imaging phenomenon that is characterized by hypointensity on T1 weighted images and hyperintensity on STIR or fat-suppressed T2 weighted images. It was first recognized as a transient increase in bone marrow water content by Wilson in 1988 in 10 patients with hip or knee pain which he suspected due to the MRI pattern. “For lack of a better term and to emphasize the generic character of the condition”, he introduced the term “transient marrow edema syndrome” [1].

Currently, various pathologies of the bone are known that can lead to a secondary, reactionary bone marrow edema. Triggering pathologies can be traumatic (stress fracture, contusion, post-surgical), septic (osteomyelitis, septic arthritis), inflammatory (enthesitis), degenerative (osteoarthritis), neoplastic (benign or malignant bone tumors), ischemic/neurogenic (avascular necrosis, Charcot neuro-osteo-arthopathy) or metabolic [2]. In addition to these known causes, some patients with nonspecific subacute or chronic joint pain and the radiologic diagnosis of bone marrow edema may not have any known underlying pathology. In these cases, the term “bone marrow edema syndrome” (BMES) was used and the disease was recognized as a separate entity [3,4]. Various synonyms have been used to describe this disease. These terms are often stated in a confusing and inconsistent manner in the literature including “transient osteoporosis” (of the hip), “regional osteoporosis” (regional), “migratory osteoporosis”, “transient bone marrow edema” and “bone marrow edema syndrome”, to name the most common. The term “transient osteoporosis” was first used by Curtiss and Kincaid in 1959 [5]. This descriptive term of the pre-MRI-era was used for the affected joints of pregnant women, showing radiolucency interpreted as osteopenia on conventional radiographs [5].

BMES is described as a self-imitating disease with a spontaneous onset of debilitating joint pain, usually of the lower extremity, which worsens with weight bearing [3,6,7]. It is a rare disease, affecting mostly middle-aged men between 40 and 60 years, but epidemiological data are lacking [3,6,8]. The etiology and pathophysiology remain largely unknown [2,3].

Resent reports highlight the role of vitamins (vitamin K, B, C) in skeletal health and on bone mineral density [9,10,11]. Especially the role of vitamin D in osteoporosis, in bone metabolism and in the health of the bone microenvironment has often been demonstrated in the past [12]. This suggests a possible relationship between vitamin status and BMES. Therefore, the aim of this scoping review was to systematically evaluate and combine the available evidence about vitamins and BMES.

## 2. Materials and Methods

Protocol and registration: The scoping review was conducted using the PRISMA guidelines and the recommended checklist (Preferred Reporting Items for Systematic Reviews and Meta-Analyses extension for Scoping Reviews; PRISMA-ScR; http://prisma-statement.org (accessed on 29 April 2022)). The scoping review was registered prior to data analysis on the platform “Open Science Framework” under the registration number 42HGX (https://doi.org/10.17605/OSF.IO/42HGX (accessed on 25 September 2022)).

Eligibility criteria: For this scoping review, we included published peer-reviewed journal papers that were written in English or German, included human participants and addressed vitamins in combination with BMES. Review articles and meta-analysis were excluded as research sources. Moreover, we excluded manuscripts that analyzed patients with secondary bone marrow edema, including osteochondral lesions, osteonecrosis, fractures, any kind of trauma and diabetic foot syndrome.

Information sources and search: To identify suitable articles for the scoping review, a database search was conducted. The database search was carried out on three databases (OVID, Pubmed and Web of Science). The search was drafted by the last author and further refined through team discussion. The search string we used was: “((migratory-osteoporosis) OR (bone-marrow-edema) OR (bone-marrow-oedema) OR (migratory-osteopenia) OR (transient-osteopenia) OR (idiopathic-osteoporosis) OR (idiopathic-osteopenia) OR (transient-osteoporosis)) AND (vitamin)”. The search was performed on 29 April 2022, with a final update of the records identified on 25 September 2022. Duplicates were identified and excluded using Endnote 20 (Cleverbridge GmbH; Köln, Germany).

Selection of sources of evidence: Three authors screened all articles (title and abstract) for the predefined eligibility criteria. A manuscript was considered suitable for further analysis if two of the three authors (AE, ME, IS) agreed that the manuscript met the eligibility criteria (primary literature). In the next step, a full text analysis was performed, again considering the eligibility criteria. Additionally, we screened the literature of the finally eligible articles for relevant references. Reference articles were included in the final analysis if their core-statement was associated with eligibility criteria (secondary literature). Full texts were included in the quantitative synthesis, systematically analyzed and evaluated by the authors for the purpose of the scoping review.

Data charting process, data items, synthesis of results: Each full text was carefully read by two authors. The information contained in the manuscript was extracted, discussed and put into tabular form. We abstracted data into manuscript characteristics (country of origin, number of patients, gender, study type), levels of vitamin, affected bones, follow-up period, therapy and time to achieve therapeutic success. Finally, we grouped the studies by epidemiology, localization, bone mineral density measurement, laboratory analysis and therapy.

## 3. Results

**Figure 1:**
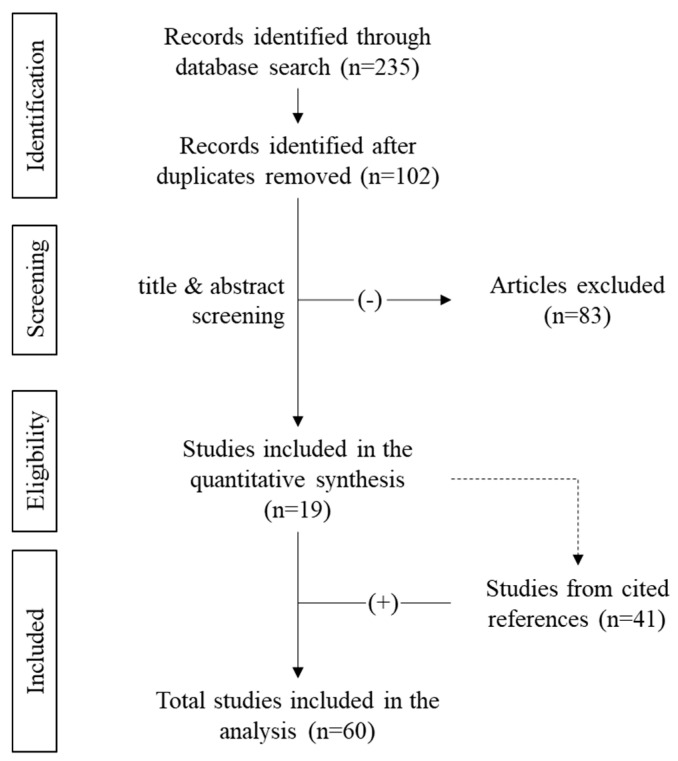
Study selection for review: Illustration of literature search and selection with numbers of articles at each stage.

Selection of sources of evidence: From the three database searches, we identified 235 articles. Upon removal of the duplicates, 102 papers remained. After application of the eligibility criteria, 19 relevant articles (primary literature) were identified for further analysis. For the primary literature, nine papers were case reports (1 patient per case report), eight papers were retrospective case series or retrospective observational studies (19.5 patients per study), one prospective observational study (12 patients) and one retrospective cross-sectional study (65 patients). The manuscripts of the primary literature were very heterogeneous and of diverging scientific scope. A synopsis of the primary literature would not be sufficient due to the limited evidence provided by the manuscripts. Therefore, we expanded the eligibility criteria to the references of the primary literature (secondary literature). Thus, an additional 41 papers could be identified and added into our scoping review. The total number of analyzed manuscripts was 60 (Figure 1).

Bibliographic characteristics: All 60 studies are composed of 28 (47%) case reports, 18 (30%) retrospective studies, 11 (18%) prospective studies, 2 (3%) longitudinal studies and 1 (2%) randomized controlled phase III trial. The included total number of patients are 37 for the case reports (1.3 patients per report), 443 for the retrospective studies (24.6 patients per study), 207 for the prospective studies (18.8 patients per study), 88 for the longitudinal studies (44.0 patients per study) and 48 for the randomized controlled phase III trial. Most of the studies were conducted in Germanophone countries (37% in Germany, Austria and Switzerland) followed by Italy (13%), USA (10%), Spain (5%) and Greece (5%).

Epidemiology: Altogether, 823 patients are included in all studies. The distribution of males to females is 1.55 ♂/1♀. Most of the included studies do not focus on a specific age group. Only six studies/case reports exclusively focus on BMES in children, although children were included in several studies. The mean age of all patients is 40.9 years. The youngest patient observed with BMES is seven years old, the oldest 79 years old. Nine patients were pregnant during onset of symptoms or while being diagnosed with BMES. The mean age of patients included in case reports is 36.1 years. In the case reports, more female patients were reported (*n* = 21) than male patients (*n* = 14).

Localization: Bone marrow edema syndrome almost exclusively affects the weight-bearing regions of the body. In all 60 included articles, 296 patients suffered from BME of the proximal femur, making the hip joint the most commonly affected site (Table 1). Most of the remaining cases are shared between the other two big weight-bearing joint regions, namely the foot and ankle (244 patients) and the knee (198 patients). Few cases could be observed at the weight-bearing axial skeleton, i.e., the pelvis (os sacrum, os pubis, acetabulum; 15 patients) and the lumbar spine (3 patients). Only three cases were reported at the upper extremities: two at the hand and one at the sternum. Interestingly, both cases affecting the hand were observed in children [13,14], with one due to vitamin C deficiency. Of the 823 patients, 151 suffered from multifocal BMES, which means the involvement of two or more joints, mostly bilateral BMES of the hip or more than one affected bone at the same region. As a result, altogether 1146 bones were affected. Multiple bone involvement mostly concerned the foot and ankle with 530 bones in 244 patients. At the foot and ankle, the talus was the most affected bone (*n* = 145), followed by the tarsus (os naviculare, os cuboideum, ossa cuneiforma; *n* = 111), the calcaneus (*n* = 57), metatarsals (*n* = 47) and the distal part of the tibia (*n* = 27).

Vitamin levels: The vitamin D levels of patients with BMES were reported in 16 studies (223 patients). Ten studies were conducted in northern Europe and the USA. According to the definition of the Endocrine Society, 105 patients (47.1%) had a vitamin D deficiency (<20 ng/mL), 40 patients (17.9%) had a vitamin D insufficiency (21–29 ng/mL) and 78 patients (35.0%) had normal vitamin D values (>30 ng/mL). Three case reports (1 patient each publication) associated vitamin C with BMES. In all cases, the patients had reduced serum levels of vitamin C due to malnutrition or restrictive eating habits and were successfully treated with vitamin C supplementation.

Bone mineral density measurement: 15 of 60 articles reported bone mineral density (BMD) measurements, but of the 232 patients included in those articles, only 148 received BMD measurements. Dual energy X-ray absorptiometry (DXA) of the lumbal spine and proximal femur was the most used BMD test, being used in 142 patients. Other BMD tests included high-resolution quantitative computed tomography (HR-pQCT) of the distal radius and distal tibia, as well as quantitative ultrasound of the calcaneus. Seven articles are case reports with a maximum of three patients, the remaining eight articles include ten or more patients with BMD measurements; the largest cohort was reported by Oehler et al. [7] with 57 DXA and 37 HR-pQCT measurements. Thus, data about BMD in patients with BMES is rare. Results of BMD measurements are reported as T- and Z-scores, absolute BMD values or categories (“normal”, “osteopenic” or “osteoporotic”), making comparisons between studies difficult. If categorized according to the WHO definition of osteopenia (T-score −2.5 to −1) and osteoporosis (T-score < 2.5), 126 of the reported patients are classifiable: 35.7% of patients with BMES have normal BMD, 38.9% are osteopenic and 25.4% suffer from osteoporosis. Varenna et al. report on 16 patients with BMES of the hip who received DXA measurements of the affected as well as the unaffected hip. BMD of the unaffected hip showed normal values according to age and gender, whereas the involved hip had a significant median decrease in BMD: 16.6% for the total hip and 22.5% for the femoral neck. This finding supports the term of “transient regional osteoporosis” as a synonym for BMES. During therapy, BMD increases significantly after 2 and 4 months in the affected hips [15]. An increase of BMD or a decrease in the difference between affected and unaffected hip after therapy is confirmed by other authors [16,17,18,19].

Therapy: Apart from three studies [7,20,21], all articles report on therapies of BMES, but among all remaining 57 articles, only one randomized, controlled phase III trial (level of evidence I) exists [22]. Most other are case reports, retrospective case series and few observational studies, mostly retrospective. Therefore, evidence on therapies and their efficiency in BMES is weak. Among the different therapy strategies, nearly all authors report on reducing weight of the affected extremity, reaching from bed rest to non-weight-bearing (NWB), partial weight-bearing (PWB) and avoiding intense activities. Another common component of therapy is the substitution of vitamin D and calcium. Most authors report on dosages of at least 800 IU vitamin D and 600 to 1000 mg calcium per day. To systematically analyze the treatment options for the BMES, we grouped the therapeutic approaches into “conservative”, “osteoactive drugs”, “iloprost” and “others”. All studies with their therapeutic regimen and results are summarized in Table 2.

Conservative therapy: In this group, therapies are summarized which consist of a combination of limited weight-bearing, analgesics, mostly NSAIDs, physical therapy and vitamin D substitution; 13 articles can be assigned to this group, and among those are 10 case reports with three or fewer patients. Pieropan et al., Orr et al. and Radke et al. [23,24,25] report on 13, 14 and 10 patients, respectively, in a retrospective, observational way. Duration of therapy mostly lasts from 3 to 6 months or until the patient is asymptomatic. Results are reported by MRI control and clinically without objectivation by scores. Healing is described to take between weeks and 1 year, mostly around 3 to 6 months.

Osteoactive drugs: Osteoactive drugs include bisphosphonates and teriparatide, a recombinant form of parathyroid hormone, and 22 reports can be assigned to this group; 19 about bisphosphonates, including those studies with the highest number of patients and the highest level of evidence. Alendronate, ibandronate, zolendronate, pamidronate, neridronate and clodronate are the bisphosphonates being used. Three articles only indicate “bisphosphonates” without specification. Therapy regimens differ from a mid-term oral application, namely alendronate 70 mg/week for 1 to 6 months, a single i.v./s.c. application (zolendronate, teriparatide) or a short-term i.v. application (ibandronate, 1–3 times). Success of therapy is mostly controlled clinically and by MRI; healing time differs, mostly between 1 and 6 months with a peak around 2 and 3 months.

Iloprost: The use of iloprost, a vasodilating prostacyclin analogon, which is used for thromangiitis obliterans, pulmonal hypertonia and M. Raynaud, is reported in 10 articles. Among them, five are from the same working group [26,27,28,29,30]. The application is i.v., mostly on 5 consecutive days, in a dosage of 20 to 50 µg/d. The occurrence of side effect is common, apparently depending on dosage. Nevertheless, most studies report a quick decrease in pain levels within the first days of treatment with an overall healing time from weeks to 3 months.

Others: Further therapy regimens of BMES include calcitonin, vitamin C substitution, core decompression and extracorporal shock wave therapy. Vitamin C substitution is described in three case reports [13,31,32], all of them with an etiology of proven vitamin C deficiency. All authors report positive results of their therapies, but altogether, the number of reports and cases is small (s. Table 2).

Comparative studies: In eight articles, different therapy forms are compared. The only RCT by Seefried et al. compares the use of zolendronate plus PWB and vitamin D/calcium substitution to placebo and PWB, vitamin D/calcium. A significant difference in the edema volume reduction in favor of zolendronate is stated, as well as a significant reduction of pain level after 3 and 6 weeks by zolendronate [22]. Bartl et al. conducted a prospective observational study, comparing ibandronate to a conservative regimen [33]. Both therapy forms could significantly reduce pain after 6 and 12 months with a better reduction by ibandronate as well as a significant (ibandronate) versus a non-significant (conservative) edema volume reduction after 6 months.

## 4. Discussion

In this scoping review about BMES and vitamins, 19 sources could be identified by primary research and an additional number of 41 by secondary research. Thus, a total number of 823 patients was included. The overall evidence is poor, especially concerning the role of vitamins in BMES: 223 of the included patients had vitamin D analysis and only 3 patients were associated with vitamin C deficiency. Additionally, the study quality is poor with 47% of the included articles being case reports.

Our review shows, that the prevalence of vitamin D deficiency and insufficiency among patients with BMES is high with 47.1% and 17.9%, respectively, and thus only 35.0% of patients with normal vitamin D values. This is even more striking, considering the young patient’s average age of 40.6 years. Therefore, a pathophysiological relationship between vitamin D deficiency and BMES seems likely, but all included studies reporting vitamin D status are lacking an age-, sex- and seasonal-matched control group. Almost all studies were conducted on the northern hemisphere, namely central Europe and North America. Vitamin D deficiency is known to be of a high prevalence in those countries [34,35,36]. In a recent survey, Rabenberg et al. report 32.8% of vitamin D deficiency and 28.7% insufficiency in men aged between 18 and 44 years in Germany and 28.9% of vitamin D deficiency and 27.0% of insufficiency for women in the same age group [34]. Furthermore, vitamin D levels are known to highly depend on season and latitude as well as other factors like age, BMI, sports activity and media consumption [34,35,36]. Therefore, it is difficult to conclude a causal relationship between vitamin D deficiency and BMES without a reference population.

Similar results could be observed in this review regarding bone mineral density. The prevalence of osteopenia and osteoporosis was high among the study population, with 38.9% (osteopenia) and 25.4% (osteoporosis), but only 148 of all 823 patients received BMD measurements. A transfer of those results to the general population suffering from BMES is therefore problematic as some authors report on selection of patients for BMD measurements. Karantanas et al. state that only men > 70 years and women > 60 years were indicated to DXA, and according to Singh et al., only patients with “risk factors” received BMD tests [37,38]. Thus, a selection bias with overestimation of the prevalence of osteoporosis is possible. Furthermore, analogous to vitamin D levels, no control groups exist to distinguish between co-existing osteopenia/osteoporosis due to general prevalence and causal osteoporosis.

Regarding the role of vitamin C in BMES, three case report were available. All patients suffered not only from BME but also from other symptoms of scurvy due to severe vitamin C deficiency because of their eating habits (malnutrition due to alcoholism, anorexia nervosa, diet lacking fresh fruit and vegetables) [13,31,32]. Because vitamin C deficiency is a well-known cause of scurvy, it is reasonable to assume that there could be a causal correlation between scurvy and bone marrow edema. It is still not clear if, in those cases, the patients suffered from a secondary bone marrow edema due to scurvy or had a “bone marrow edema syndrome” in its classical meaning. A recent review by Rowe et al. highlights, that vitamin C deficiency is common in low- and middle-income countries and still existent in high-income countries [39]. The prevalence in an age group comparable to this review ranges between 1% (France [40,41]), 1.4% (England [42,43]), <3% (Canada [44]) and 8.4% (USA [45]). Apart from the abovementioned case reports, vitamin C levels in patients suffering from primary BMES have not been published yet. It would be interesting and a possible focus for further research if the prevalence of vitamin C deficiency in patients with BMES is higher than in the general population and if there is a pathophysiologic relationship.

As the pathophysiology of BMES remains largely unknown, no causal therapy exists. The best evidence is available for bisphosphonates and iloprost therapy, but the overall evidence is poor due to lacking RCTs. Vitamin D substitution is common in many therapy protocols, again assuming a pathophysiologic relationship, but no study compares vitamin D substitution against no substitution. Reducing weight and vitamin D substitution appear in a plethora of combinations among other therapeutically measures in most studies, so that the role of those measures is not evaluable within the existing data.

All studies report on successful treatment of BMES. Publication bias may be one cause, but additionally, as BMES is considered a self-limiting disease, the influence of therapy is difficult to demarcate against spontaneous healing when lacking control groups. When comparing healing times, the time between onset of symptoms and beginning of therapy has to be considered, as this time is already part of the spontaneous healing time. As the analyzed studies are heterogenous and poor in quality, a meta-analysis for therapies is not feasible.

This review has a few limitations. As the primary search strategy focused on the literature about BMES and vitamins, the secondary literature might not be complete, especially regarding therapies. Furthermore, we did not focus on subgroups such as pregnancy-associated bone marrow edema or growth-associated BME in children as it is still unclear whether they represent a separate entity or are part of bone marrow edema syndrome. As the definition and nomenclature of BMES is sometimes challenging and some authors do not clearly define the origin of the BME they report on, it is possible that secondary BME is included in this analysis.

**Table 2:** Summary of all studies.

Author	Year	Country	Study Type	*n*	Therapy	Duration of Therapy	Result Clinically	Result Radiologically (MRI)	Healing Time	Follow Up Time
Conservative										
Alsaed O. [46]	2018	Qatar	CR	1	Avoid prolonged WB, NSAID, Vit. D	3 m	asymptomatic		“few weeks”	3 m
Kaspiris A. [47]	2019	Greece	CR	1	Avoid WB, NSAID, Vit.D	6 m	asymptomatic	6 months: normal	6 m	6 m
Pieropan S. [23]	2019	Italy	RCS	13	Avoid intense activity, analgetics, PT, Vit. D., cortisone p.o.	3 m	asymptomatic		3 m	3 m
Orr, JD. [24]	2010	USA	RCS	14	PWB ± cast, NSAID, PT,		asymptomatic (86%)	4/14: 75% normal	4 m (up to 18 m)	20.7 (3.5–43) m
Axt-Fliedner R. [48]	2001	Germany	CR	1	Bedrest, PWB, analgetics, PT	n.r.	improved		post partum	n.r.
Daniel RS. [49]	2009	USA	CR	1	analgetics	n.r.	n.r.	12 months: reduction	12 m	12 m
Diwanji SR. [18]	2008	Korea	CR	2	NWB, NSAID, PT	n.r.	asymptomatic	9 months: normal	3–7 m	10–22 m
Escolà A. [50]	2009	Spain	CR	1	PWB, deflazacort	n.r.	asymptomatic	6 months: normal	3 m	12 m
García Garzón JR [51]	2005	Spain	CR	1	“conservative treatment”	n.r.	asymptomatic		5 m	5 m
Joshi V. [52]	2014	USA	CR	1	limit intense activity,	n.r.	asymptomatic	4 months: normal	4 m	8 m
Palit G. [53]	2006	Belgium	CR	1	Avoid physical activity, paracetamol	n.r.	asymptomatic		6 w post partum	n.r.
Radke S. [54]	2001	Germany	PCS	10	PWB, analgetics 4/10: core decompression after 1–2 months	n.r.	Weber ankle score improvement from 52 to 95		3–9 m CD: 3–12 m	12 m
Yamasaki S. [55]	2003	Japan	CR	3	PWB, NSAID	n.r.	asymptomatic	2–8 months: normal	2–8 m	n.r.
Osteoactive Drugs										
Emad Y. [56]	2021	Egypt	CR	1	Alendronate 70 mg/week p.o. PWB, Vit. D, calcium	6 m	improvement	3 months: almost total regression	3 m	3 m
Emad Y. [57]	2012	Egypt	PCS	8	Alendronate 70 mg/week p.o. NWB, Vit. D, calcium	6 m	n.r.	6 months: normal	6 m	6 m
Evangelatos G. [58]	2019	Greece	RCS	9	Alendronate 5 mg i.v. single PWB, Vit. D, calcium	1 m	asymptomatic (100%)	3 months: 100% normal	1.1 ± 0.5 m	60.7 ± 34.4 m
Karantanas AH. [37]	2008	Greece	LDS	63 *	Alendronate 10 mg/d p.o. NWB, NSAID, calcium	3 m	improvement (100%)		8.4 (±3.9) m	3 y
Kibbi L. [59]	2007	USA	CR	3	Alendronate 70 mg/week p.o.	5 m	asymptomatic	5 months: normal	3 w–2 m	n.r.
Miltner O. [60]	2003	Germany	CR	1	Alendronate 70 mg/week p.o. NWB, Vit. D., calcium	3 m	asymptomatic	3 months: normal	9 w	3 m
Simon MJ. [61]	2014	Germany	RCS	25	Ibandronate 3 mg i.v. single; 2nd/3rd application after 4–6 weeks optional PWB, Vit. D, calcium		pain reduction after 2 weeks: 64% return to competition time 102.6 ± 65.2 d	n.r.	102.6 ± 65.2 d	395 ± 269.7 d
Ringe JD [16]	2005	Germany	POS	12	Ibandronate 4 mg i.v. single; 2 mg ibandronate after 3 months optional Vit. D, calcium		decrease in pain score 43.3% (1 month), 78.4% (3 months), 94.3% (6 months)	DXA: increase lumbar spine BMD 4%		6 m
Carty S. [62]	2007	Great Britain	CR	2	Pamidronate 60 mg i.v. single		asymptomtic	1 months: normal/reduced	2–4 w	n.r.
Varenna M. [15]	2002	Italy	PCS	16	Pamidronate 45 mg i.v. 3× every 3rd day		asymptomatic: 43.8% (1 month), 87.5% (2 months); significant improvement VAS and FUI score after 1 month	3 months: normal	1–2 m	39.5 ± 17.7 m
La Montagna G. [63]	2005	Italy	CR	1	Neridronate 25 mg/month i.m. Bed rest, Vit. D, calcium	6 m	asymptomatic	4 months: normal	2 m	4 m
Trevisan C. [64]	2002	Italy	CR	3	Clodronate 100 mg/d i.v., Calcitonin 100 U/d NWB, NSAID, PT	2–6 m	asymptomatic		1–10 m	
Sprinchorn AE. [65]	2011	Australia	RCS	10	“Bisphosphonates” Walker, Vit. D, calcium	5–10 m	asymptomatic		5–10 m	13 (4–21) m
Vaishya R. [66]	2017	India	RCS	12	“Bisphosphonates” PWB, NSAID, Vit. D, calcium	n.r.	asymptomatic		17.1 (13–25) w	1.3 (1–2.5) y
Trevisan C. [67]	2016	Italy	ROCS	23	“Bisphosphonates” PWB	n.r.	“healing”		4.3 (2–7) m	n.r.
Rolvien T. [68]	2017	Germany	ROS	14	Teriparatide 60mg s.c. single Vit. D; 2/14 + Core decompression		significant decrease (VAS)	6–12 weeks: completely resolved: 50%, reduced: 43%, constant: 7%	6–12 w	n.r.
Fabbriciani G. [19]	2010	Italy	CR	1	Teriparatide20 µg/d PWB, Vit. D	1 m	asymptomatic	2 months: almost normal	1 m	n.r.
Geith T. [69]	2015	Germany	CR	1	Teriparatide 60 mg s.c. single		asymptomatic	2 months: normal	2 m	2 m
Iloprost										
Aigner N. [29]	2001	Austria	PCS	6	Iloprost 50 µg i.v. on 5 consecutive days		Mazur score improvement from 58 to 91 (1 month), 93 (3 months)	3 months: normal (100%)	5 (3–6) w	6 m
Aigner N. [26]	2003	Austria	PCS	19	Iloprost 50 µg (6/19), Iloprost 20 µg (13/19) i.v. on 5 consecutive days PWB if needed		pain relief: 79%; non-responder: 21%; Mazur score improvement from 54.9 to 87.8	3 months: 63% completely resolved, 16% subtotal regression, 21% no improvement		3 m
Aigner N. [27]	2002	Austria	CR	1	Iloprost 20 µg i.v. on 5 consecutive days		asymptomatic	6 weeks: almost complete resolution 12 weeks: normal	2 w	6 m
Arazi M. [70]	2006	Turkey	CR	1	Iloprost 40 µg i.v. on 5 consecutive days		asymptomatic	3 months: nearly complete resolution	3 m	2.5 y
Hörterer H. [71]	2018	Germany	OCS	11 **	Iloprost 20 µg dL, 40 µg d2–5 WB as tolerated, Analgetics, PT		3 months: 56% pain decrease, 38% no pain relief	3 months: 83% considerable decrease		28 ± 19 m
Meizer R. [30]	2005	Austria	ROS	27 ***	IIloprost 20–50 µg/d on 5 consecutive days PWB		4 months: 75.2% pain decrease	4 months: 92.6% significant improvement	4 m	4 m
Tosun HB. [72]	2020	Turkey	RCS	23	Iloprost 20 µg i.v. on 5 consecutive days, Alendronate 70 mg/week, FWB, ASS 150 mg, Vit. D, calcium	3 m	significant reduction VAS after 3 and 6 months (from 9.1 to 2.4), significant improvement FMS score	3 months: 60.9% complete regression, 17.4% minimal edema, 21.7% moderate edema		n.r.
Comparative Studies										
Seefried L. [22]	2022	Germany	RCT	48	(1) Zolendronate 5 mg i.v. single; PWB, Vit. D, calcium (2) Placebo; PWB, Vit. D, calcium		(1) significant pain reduction week 3 and 6 (2) non-significant reduction week 3 and worsening week 6	6 weeks: volume reduction from (1) 69.7 cm³ to 25.2 cm³ (2) 44.0 cm³ to 19.6 cm³ Significant difference between groups; Decrease/complete resolution of edema (1) 76.5%/38.2% (2) 50%/21.4%		3 m
Pabinger C. [73]	2012	Austria	CR	1	(1) NWB, PT (2) Iloprost 300 µg/d for 5 d, followed by pamidronate 30 mg/d for 3 days, Vit. D, calcium	(1) 3 m (2) 3 m	(1) asymptomatic (2) asymptomatic	(2) normal	(1) 3 m (2) 2 w	10 m
Bartl C. [33]	2012	Germany	POS	50	(1) ibandronate 6mg i.v./months 3× (n = 30) (2) 3 weeks NWB, 3 weeks PWB, NSAID (n = 20)	(1) 3 m	VAS decrease from (1) 8.5 to 1.6 (6 months) and 1.2 (12 months) (2) 8.1 to 4.6 (6 months) and 4.0 (12 months) Scores: significant improvement in both groups (6 + 12 months); Ibandronate vs. Conservative sign. better	6 months: (1) significant reduction (2) non significant reduction	(1) 3 m (2) not defined	1 y
Radke S. [74]	2003	Germany	ROS	43	(1) PWB, NSAID (2) Core decompression, NWB	n.r.	HHS improvement from (1)38.6 to 80.6 (2) 48.5 to 77.2			2–10 y
Singh D. [38]	2015	Great Britain	ROS	18	(1) PWB alone (n = 7) (2) PWB, then Zolendronate 5 mg i.v. (n = 9) (3) PWB, then Alendronate p.o.(n = 2)				(1) 25.6 w (2) 13.8 w (3) 24.0 w	5.75 y
Muller F. [75]	2020	Switzerland	ROS	34	(1) Ibandronate i.v. multiple (n = 9) (2) Zoledronate i.v. singe (n = 12) (3) Ibandronate i.v. multiple followed by Zoledronate iv single (n = 7) (4)Teriparatide s.c. single (n = 3) (5) Alendronate p.o. (n = 3)		response to treatment (1) 89% (2) 100% (3) 86% (4) 100% (5) 100%	18/34: Edema reduction (1) 67% (2) 92% (3) 86% (4) 67% (5) 100%		n.r.
Aigner N. [28]	2005	Austria	ROS	36	(1) Iloprost 20 µg i.v. on 5 consecutive days, PWB (n = 18) (2) Core decompression, NWB (n = 20)		HHS improvement from (1) 64.7 to 97 (3 m) (2) 53.7 to 95.1 (3 m)	(1) normal (100%) (2) 70% complete remission, 20% residual edema, 10% progression to avascular necrosis	(1) 4 (0–12) w (2) 6 w	n.r.
Baier C. [76]	2012	Germany	ROS	20	(1) Iloprost 1 d 20 µg, 1 d 30 µg, 3 d 40 µg (2) Ibandronate 6 mg/month	(2) 3 m	VAS: significant improvement (1) from 6.4 to 1.1 (3 months), 1.1 (12 months) (2) from 5.6 to 2.6 (3 months), 1.5 (12 months) WOMAC: significant improvement (1) 53.6 to 13.4 and 12.1 (2) 50.5 to 27.5 and 20.8	3 months: (1) 43% complete regression, 43% reduction, 14% non- responder (2) 33% complete regression, 50% reduction, 17% non-responder	(1) first days—4 w (2) first days—3 m	12 (10–17) months
Others										
Laktasic-Zerjavic N. [77]	2007	Croatia	CR	1	Calcitonin 200 IU/d nasal; PWB, Vit. D, calcium	2 m	asymptomatic	1 y: normal	2 m	1 y
Arayssi TK. [17]	2003	Lebanon	CR	2	Calcitonin 200 IU/d sc/nasal; PWB, Vit. D, calcium	n.r.	mild symptomatic/asymptomatic	normal	6–9 w	
Fernandez-Canton OC. [78]	2003	Spain	LDS	25	Calcitonin, rest, NSAID, Vit. D, calcium	n.r.	76% asymptonatic	72% resolution, 20% partial improvement, 8% no improvement		9–13 m
Berger CE. [79]	2003	Austria	PCS	37	Core decompression		recovered (100%)	3 m: normal (100%)	6 w	12 (12–48) m
Radke S. [74]	2003	Germany	PCS	18	Core decompression; 6 weeks NW, 6 weeks PWB	12 w	pain free (100; significant HHS improvement from 37.2 to 93.7	6 m: 91% normal	7.2 (1–30) d	6 m
D‘Agostino C. [80]	2014	Italy	PCS	20	Extracorporal shock waves 2× (after 48 h), PWB	1 m	HHS: significant improvement after 2 and 3 months from 39.0 to 81.3 (2 m), 91.8 (3 m), 95.1 (6 m)	significant edema reduction after 2 and 6 m	3 m	15.5 ± 1.9 m
Amar SK. [31]	2021	Great Britain	CR	1	Vitamin C substitution		asymptomatic		3 w	3 m
Liebling EJ. [13]	2020	USA	CR	1	Vitamin C 100 mg 3×/d for 7 days, 100 mg/d	3 w	asymptomatic		3 w	3 w
Rodriguez S. [32]	2007	USA	CR	1	Vitamin C substitution, NSAID, PT					Lost to FU
Sconza C. [81]	2022	Italy	CR	1	Neridronate 100 mg i.v. 4x every 3 days, extracorporal shockwaves 3×/week, NWB, NSAID, PT, Vit. D, calcium	4 m	asymptomatic	4 m: normal	4 m	4 m
Kroger L. [14]	2004	Finland	CR	1	n.r.		asymptomatic	8 m: normal	3 m	8 m
Without therapeutical intervention										
Hadidy AM. [20]	2009	Jordan	RCS	17						
Horas K. [21]	2017	Germany	RCS	31						
Oehler N. [7]	2018	Germany	RXS	65						

Abbreviations: w: week(s); m: month(s); y: year(s); CR: case report; RCS: retrospective case series; PCS: prospective case series; POS: prospective observational study; ROS: retrospective observational study; RCT: randomized controlled trial; LDS: longitudinal study; ROCS: retrospective observational cohort study; RXS: retrospective cross-sectional study; PT: physical therapy; WB: weight bearing; PWB: partial weight bearing; NWB: non weight bearing; CD: core decompression n.r.: not reported; VAS: visual analogue scale; HHS: Harris Hip Score. * 98 patients included in study, but only 63 with BMES. ** 42 patients included in study with different etiologies of BME, only 11 patients with BMES. *** 104 patients included in study, with different etiologies of BME, only 27 patients with BMES.

## 5. Conclusions

There is limited data on BMES in association with vitamin status. A high prevalence of vitamin D deficiency and osteopenia among patients with BMES suggests a pathophysiologic connection, but a causal relationship between vitamin D status, osteopenia and BMES cannot be made within the existing literature. Vitamin C deficiency seems to be causal for BME in patients suffering from scurvy, but no data exists about the prevalence of acute or subacute vitamin C deficiency in patients suffering from primary BMES. Various different therapy regimens are successful, with bisphosphonates and iloprost as the most promising candidates in reducing healing time. For further research and better understanding of this disease, epidemiological data with a special focus on vitamin status and bone mineral density compared to a matched control population, as well as randomized, controlled trials for therapy evaluation, are needed.

## Figures and Tables

**Table 1:** Localization of the bone marrow edema, total number of patients und number of bones.

Localisation	Number of Patients	Number of Bones
Pelvis	15	20
Proximal femur	296	340
Knee	198	241
Foot/Ankle	244	530
Lumbar Spine	3	3
Hand	2	3
Sternum	1	1
Not specified	73	8
Multifocal	151	-
Total	823	1146

## Data Availability

The data generated during current study are available from the corresponding author on reasonable request.
